# Relationship between Pharmacists’ Emotional Intelligence and Job Performance: A Cross-Sectional Study in Saudi Arabia

**DOI:** 10.3390/pharmacy12050145

**Published:** 2024-09-25

**Authors:** Yasser S. Almogbel, Muath A. Alsalloum, Rubiaan S. Almadi, Abdulaziz A. Almazyad, Yusuf M. Garwan, Razan A. Alregaibah

**Affiliations:** 1 Department of Pharmacy Practice, College of Pharmacy, Qassim University, Buraydah 51452, Saudi Arabia; 2 Department of Pharmacy Practice, College of Clinical Pharmacy, Imam Abdulrahman Bin Faisal University, Dammam 34212, Saudi Arabia; maalsalloum@iau.edu.sa (M.A.A.); ymgarwan@iau.edu.sa (Y.M.G.); 3 King Faisal Specialist Hospital and Research Centre (KFSHRC), Pharmaceutical Care Division MBC 11, Riyadh 11564, Saudi Arabia; ralmadi@kfshrc.edu.sa; 4 Pharmaceutical Services, Qassim University Medical City (QUMC), Buraydah 51452, Saudi Arabia; aalmazyadd@gmail.com; 5 Department of Pharmacy Practice, King Khalid University Hospital, King Saud University, Riyadh 12372, Saudi Arabia; razanalregaibah@gmail.com

**Keywords:** emotional intelligence, job performance, pharmacists, Saudi Arabia, questionnaire, observational study, health care personnel

## Abstract

Pharmacists’ job performance is crucial for improving pharmacy services. The purpose of this study was to evaluate the association of emotional intelligence with the job performance of pharmacists in Saudi Arabia. Using social media platforms, we disseminated an online questionnaire to pharmacists licensed to practice in Saudi Arabia between June and July 2022. The questionnaire was filled out by 352 pharmacists. The majority of the participants were women (60.5%) and working as community pharmacists (55.7%). On self-reported emotional intelligence and job performance scales, the respondents scored an average of 5.5 ± 0.9 (out of 7) and 4.0 ± 0.6 (out of 5), respectively. Multiple linear regression analyses revealed that emotional intelligence had a significant relationship with job performance (β = 0.43, *p* < 0.001). In conclusion, the findings indicated that emotional intelligence may influence the job performance of pharmacists in Saudi Arabia. As the primary objective of every organization is to accomplish the best possible performance, prioritizing emotional intelligence is important. Further research is needed to identify the impact of emotional intelligence on work performance, which could potentially enhance clinical outcomes for patients.

## 1. Introduction

The ability to comprehend and control one’s emotions is referred to as emotional intelligence (EI). Previous studies have reported that EI enhances productivity in the workplace by increasing the collaborative and correspondence activities of an employee [1,2]. The continued growth and importance of EI make it a significant topic of investigation, particularly in terms of improving employee performance and determining the most qualified candidates for essential roles [2]. Ability EI and trait EI are often used to predict the EI of a person. Ability EI most often records the relationship between the cognitive skill and emotional ability, whereas trait EI suggests the self-efficacy and emotional traits [3]. Prior studies utilized both ability EI and trait EI to analyze data obtained from self-reports, personality questionnaires, and performance tests [4]. Additionally, the difference in ability EI and trait EI data can be utilized to design suitable strategies for development and accumulation of knowledge in the targeted population [5].

The four main components of EI are as follows: self-emotion appraisal (SEA), or the ability to understand one’s feelings; others’ emotion appraisal (OEA), or the capacity to grasp one’s own and others’ feelings; use of emotion (UOE); and regulation of emotion (ROE) [6]. EI has been studied in relation to the field of healthcare with the main objective of improving the services offered to patients. It has been observed that by comprehending both their own as well as patients’ emotions, emotionally intelligent healthcare professionals can increase patient safety [7]. Further, within organizational and industrial psychology, EI has gained popularity as a predictor of career success. The concept of EI has also been adopted by allied healthcare professionals and established as crucial to the success of pharmacists, as it enables them to actively listen, empathize, and control their emotions [7].

Prior studies suggested that EI is an essential component in determining the success of not only individuals but also organizations. The ability to screen one’s own and others’ sentiments and feelings, recognize them, and utilize this information to illuminate one’s reasoning and ways of behaving is a part of social insight. Adopting the concept of EI is reported to be a vital aspect of the capacity to appreciate individuals on a profound level, especially among health care providers [8,9,10]. Interactions with patients in medication counseling and addressing concerns, place pharmacists in circumstances where EI is required.

According to Medina et al. [11], pharmacists’ EI supports their ability to answer questions related to medication and health, while considering a patient’s overall situation. Competent pharmacists have the capacity to build a professional relationship to work with patients, families, and different individuals from the medical services group [12]. These abilities were also reported to support the pharmacist’s capacity to advocate for their patients’ safe medication use.

Job performance can be defined as actions or behaviors, such as quality of work, job knowledge, working relationships, and achievements, that are organization-oriented [13]. Job performance has been found to be associated with employees’ actions and behaviors they have control over, rather than to behaviors guided by the environment [14]. Improvement in job performance in the pharmacy profession can optimize patient care and minimize healthcare expenditure [15]. Pharmacists’ improved job performance can significantly reduce the time taken to administer appropriate medication, and minimize adverse drug events, emergency department visits, length of stay in intensive care units, hospital readmissions, and mortality [16,17,18,19]. Moreover, studies have indicated a statistically significant reduction in costs associated with medication use owing to pharmacists’ job performance improvement [20].

EI has been found to positively impact the job performance of healthcare professionals, including physicians, pharmacists, and nurses [21,22]. Goleman’s EI components—self-awareness, social awareness, self-management, and relationship management—are recognized to help and not only manage one’s feelings but also to respond effectively to those of others [23]. In the healthcare profession, knowledge, skills, and competencies are the core areas of the learning domain. However, empathy, EI, and inter-professional skills have been reported to play a pivotal role in executing the professional activities centered on patient care [22,24].

Furthermore, studies suggest that EI may be related to how pharmacists manage pressure, and having a certain level of EI may assist them with forestall burnout, weariness, and associated side effects [25]. Recent reports identified EI as a fundamental characteristic of effective pharmacists. The capacities and characteristics of EI—inspiration, decisive reasoning, fundamental skills associated with a pharmacist, and a balance between amusing and serious behavioral activities—were found to be related to pharmacists’ achievements [25]. Saudi Arabia, a country with a population exceeding 36 million, has an extensive healthcare network [26]. However, patients availing themselves of these facilities often complain of inadequate quality of healthcare delivery in addition to the unavailability of medicines in outpatient pharmacies and lack of medication counseling [27]. Hence, EI is a crucial factor influencing job performance, especially among pharmacists, as it enhances patient interactions, fosters teamwork, improves stress management, and supports effective leadership.

According to Joseph et al. (2015), trait EI is considered one of the known predictors for assessing job performance. Self-reporting adopted in trait EI is reported to predict the job performance of a person beyond cognitive ability [28]. Prior studies assessing EI among pharmacists also utilized trait EI as a predictor of job performance [7,29].

To our knowledge, the effect of EI on job performance among pharmacists in Saudi Arabia has not been explored. Based on the literature, we anticipate finding a significant association between EI and job performance. Therefore, this study aims to determine whether a significant correlation exists between trait EI and job performance among Saudi pharmacists.

## 2. Methods

### 2.1. Study Design and Participants

This study employed a cross-sectional design and utilized a self-administered online survey, which was validated for its structure, face, and content validity, and tested through a pilot study. The questionnaire was disseminated through social media platforms between June and July 2022.

### 2.2. Participants and Sample Size

Data were collected from pharmacists holding a valid practicing license in Saudi Arabia utilizing a convenience sampling technique. Using G*Power software v.3.1, a minimum sample size of 257 participants was calculated, based on an alpha level of 0.05, an effect size of 0.15, a power of 0.99, and 15 independent factors [30]. Although the required sample size was 257, a total of 352 surveys were collected. Incomplete questionnaires were excluded from the data analysis.

### 2.3. Survey Design

The questionnaire was divided into three main sections designed to collect data on demographics, EI, and job performance. The measurement of EI was conducted using 16 items from Wong and Law’s Emotional Intelligence Scale [8], which cover the four main aspects of EI (i.e., SEA, OEA, UOE, and ROE) [31]. Respondents rated each item on a Likert scale ranging from 1 (“strongly disagree”) to 7 (“strongly agree”). The resulting scores were then employed to compute the individual’s overall emotional intelligence and scores for each specific dimension. The EI score, representing the aggregate of the four-dimension scores, spans from 16 (minimum) to 112 (maximum). A high score on the Emotional Intelligence Scale developed by Wong and Law suggests a heightened level of emotional intelligence. Job performance was assessed using 16 items from Koopman et al.’s Individual Work Performance Questionnaire (IWPQ) [32]. The IWPQ encompasses three core domains and 18 items. The domains include (1) task performance, (2) contextual performance, and (3) counterproductive work behavior. The IWPQ commonly utilizes a 5-point Likert scale, where respondents can indicate the frequency of their engagement in specific behaviors using a numerical rating system (1 = seldom to 5 = always).

The questionnaire underwent a translation process involving initial translation from English to Arabic, followed by back-translation to ensure accuracy and consistency of meaning [33]. It was pretested on a pilot sample of 10 pharmacists to ensure clarity.

### 2.4. Statistical Analysis

Data were analyzed using the Stata Statistical Package, version 16. Descriptive and inferential statistical analyses were performed. The mean ± standard deviation and median were used to summarize continuous variables. Estimates of associations were obtained using univariate and multivariate regression analyses. A *p*-value of less than 0.05 was considered statistically significant.

### 2.5. Ethical Considerations

The study protocol was reviewed and approved by the Ministry of Health’s Qassim Province Regional Research Ethics Committee (No. 607-43-6834). Informed consent was obtained from participants prior to beginning the questionnaire. Participation was voluntary, and respondents were permitted to withdraw at any time without providing a reason. Anonymity and confidentiality were maintained, with no identifying information collected.

## 3. Results

A total of 352 pharmacists completed the survey, and their responses were included in the analysis. Table 1 and Table 2 summarize the sociodemographic characteristics of the study cohort. More than half of the respondents (60.5%) were women, with a mean age of 28 years. Most respondents reported that community pharmacies (55.7%) were their primary job setting, followed by hospital pharmacies (22.2%). These results correspond with the fact that the majority of pharmacy employment opportunities in Saudi Arabia are concentrated in these two areas. The participants’ average number of years of experience and weekly working hours were 3 ± 3.9 years and 45.3 ± 5.5 h, respectively. The mean monthly income was calculated to be 11,440 Saudi riyals, with 1 USD being equivalent to 3.75 Saudi riyals. A minor variation, which could be attributed to variance in the participants’ work settings, work hours, and years of experience, was noted.

Table 3 depicts the descriptive statistics and the reliability of the scales utilized in this study. The respondents scored an average of 5.5 ± 0.9 (out of 7) and 4.0 ± 0.6 (out of 5) on the EI and job performance scales, respectively. This resulted in an internal validity (i.e., Cronbach’s alpha value) above the established threshold of 0.70.

Univariate and multivariate linear regression analyses were performed, and the results are depicted in Table 4 and Table 5. In the univariate regression analysis (Table 4), a significant regression equation was found between job performance and EI (R^2^ = 0.33, F(1, 350) = 173.7, *p* < 0.001). A positive association was found between job performance and EI (β = 0.58; 95%, *p* < 0.001). In the multivariate regression analysis (Table 5), a significant regression equation was found between job performance and the added independent factors (R^2^ = 0.34, F(4, 347) = 44.07, *p* < 0.001). EI was found to have a significant positive association with job performance (β = 0.57, *p* < 0.001). However, in both the univariate and multivariate regression analyses, age, gender, marital status, and experience were not associated with pharmacists’ job performance.

Table 6 shows the correlations between job performance and each EI domain. All correlations between job performance and the EI domains were statistically significant (*p* < 0.001). The correlation with the SEA domain had an r = 0.42; the correlation with the UOE domain had an r = 0.62; the correlation with the ROE domain had an r = 0.62; and the correlation with the OEA domain had an r = 0.31.

## 4. Discussion

This is the first study conducted to assess the relationship between EI and job performance of pharmacists in Saudi Arabia and the Middle East. The data suggested a possible association between job performance and each of the four EI domains—the higher the EI, the higher the job performance. These findings align with those reported by Ruble et al., who evaluated a similar relationship among pharmacists licensed to practice in Florida, USA [34].

The empirical data support the notion that EI is important and could contribute to pharmacists’ job performance. The observations from this study also provide evidence that appropriately managing EI can improve pharmacists’ productivity and efficiency. These findings are compatible with those of Khalid et al., who reported the impacts of EI on organizational commitment and job performance [35]. The data from their study indicate that employees’ appraisal and evaluation of their emotions can improve their overall performance. Similarly, understanding the emotions of supervisors, coworkers, and subordinates was found to assist employees to build a progressive working environment [36].

Most respondents in this study reported working in community or hospital pharmacy settings, which is consistent with the findings of Ruble et al. [34]. This study also utilized the self-reporting (trait) method to predict the impact of EI on job performance. Moreover, the self-reporting method adopted in the present study was found to have its own advantages. The literature review indicated that EI recorded from self-reporting studies measures the perception of the targeted population and is a strong indicator of the correlation between EI and job performance [37]. Studies involving ‘other-reporting’ methodology, although they could provide information on how others perceive someone, can contribute to ‘bias’ since they do not record the EI of a person [38].

A study by AlRuthia et al. on the distribution of the licensed pharmacist workforce in Saudi Arabia reported that the majority were employed in community pharmacy settings (34.5%, *n* = 24,395) [39]. These findings are aligned with the job opportunities available for pharmacy graduates in Saudi Arabia listed in the statistical yearbook published by the Saudi Ministry of Health [40].

EI has a direct positive association with job performance, playing a crucial role in the pharmacy profession, which relies on pharmacists’ emotional resilience, teamwork, and ability to plan, monitor, and assess situations from different perspectives, which are all the key aspects of EI and job performance [41,42,43]. We observed statistically significant correlations were observed between job performance and each EI domain (*p* < 0.001), with UOE showing the highest correlation (0.62) and OEA the lowest (0.31). Despite these disparities, the variation between domains was minimal. All the Cronbach’s alpha values for the domains exceeded 0.7, which is considered acceptable for the four questions in each domain. These findings are consistent with earlier research showing that EI can enhance innovation, productivity, and job performance among pharmacists. Prior studies also suggested that pharmacists exhibit strength across all EI domains compared to other healthcare practitioners [44,45].

A prior study assessing the impact of EI on the nursing profession in Saudi Arabia revealed a moderately strong positive correlation between EI and job performance [46]. Codier et al. found a similar correlation among staff nurses in the United States [47]. The Cronbach’s alpha values observed in the current study were 0.89 for EI and 0.91 for job performance (Table 3), indicating the reliability of the questionnaire-based assessment [48]. Studies have suggested that demographic characteristics such as age, gender, and years of professional experience can influence EI [49]. In our study, the pharmacists’ average age was 28 (±3.6) years. As reported in the literature, younger individuals tend to perform well when assigned multiple tasks related to the job being assigned [50]. Moreover, the presence of a higher proportion of female participants can be linked to the sample’s better EI and job performance (Table 1). Prior research indicated that women often display greater empathy and EI in professional settings [51]. Therefore, the positive correlation between EI and job performance observed in our study could be associated with demographic characteristics such as age and gender.

A study conducted in Pakistan revealed that EI is essential for healthcare practitioners to develop interpersonal and professional abilities. As the EI of healthcare workers improves, so does their job performance, ultimately leading to increased job satisfaction [52]. Additionally, the findings of that study suggest that pharmacists may possess better social skills and higher motivation than other healthcare specialists, contributing to further improvements in job performance.

The present study has some limitations. First, owing to the cross-sectional design, causal inferences cannot be drawn. Second, the use of convenience sampling and self-reporting limits the generalizability of the findings [53]. Subjective self-assessment methods may be affected by social desirability, recall bias, and lack of self-awareness. Response fatigue can occur during lengthy or frequent surveys, leading to rushed or less thoughtful answers. Individuals may provide varied responses to the same question because of their unique perspectives, resulting in inconsistent answers. However, descriptive analysis and careful sample selection assisted with reducing selection bias. Furthermore, peer assessments with objective measures helped mitigate these issues. As reported in the literature, these limitations can be minimized by increasing the sample size and selecting participants from multiple sources [54]. Third, because convenience sampling was used, the potential effect of non-participants could not be evaluated. Considering these limitations, the study is being extended to include more samples from different regions and to estimate additional parameters such as ability EI, to precisely predict the impact of EI on job performance. The findings will be presented in a future article.

Based on the current findings, future research could provide deeper insights and practical applications for integrating EI development into pharmacy education and professional development programs. We propose two strategies for future research. First, with substantial evidence of a correlation between EI and job performance among pharmacists, future researchers should assess the impact of these findings on clinical outcomes. Second, although several studies discussed integrating EI into pharmacy curricula, none evaluated the impact of such interventions on graduates’ job performance [55,56,57,58]. Follow-up studies that evaluate student performance during internships are suggested to confirm the impact of such interventions. Finally, given that the findings of this study indicate the importance of EI in pharmacy practice and its association with job performance, organizations can utilize the outcomes of this study to recruit pharmacists and improve the attributes of their pharmacist employees.

## 5. Conclusions

Our findings revealed a significant positive relationship between trait EI and job performance among pharmacists in Saudi Arabia, contributing to the growing evidence on the importance of EI. These results suggest that integrating EI training into pharmacy curricula could yield valuable outcomes. Therefore, implementing training programs to enhance the emotional capabilities of pharmacists is crucial. Recognizing the importance of EI in cultivating efficient, high-performing staff is also essential. Encouraging pharmacists to participate in programs that connect emotions with action and develop other aspects of EI is beneficial. Furthermore, healthcare departments aiming to improve the performance of healthcare workers should foster knowledge of EI theory as part of their ongoing efforts to enhance professional expertise. The data on EI and its impact provide medical professional organizations with new insights into developing strategies and procedures to improve job performance and employee satisfaction. Further studies are necessary to investigate the impact of the EI–job performance association, including the role of ability EI, on the bedside performance of healthcare workers and clinical outcomes.

## Figures and Tables

**Table 1:** Categorical sociodemographic characteristics (*n* = 352).

Characteristics	Number of Participants (*n*)	%
Gender		
Men	139	39.5
Women	213	60.5
Marital status		
Married	138	39.2
Single	207	58.8
Divorced	7	2.0
Pharmacy job setting		
Hospital pharmacist	78	22.2
Academic pharmacist	7	2.0
Pharmaceutical marketing	52	14.8
Pharmaceutical industry	2	14.8
Community pharmacist	196	55.7
Pharmaceutical regulatory affairs	3	0.9

**Table 2:** Continuous sociodemographic characteristics (*n* = 352).

Characteristic	Mean (±SD)	Median
Age (years)	28 (±3.6)	27
Years of experience	3 (±3.9)	2
Weekly working hours	45.3 (±5.5)	45
Monthly income	11,440.7 (±4734.1)	11,000

**Table 3:** Descriptive statistics and reliability of the scales used in this study.

Item	Mean ± SD	Cronbach’s Alpha
Emotional intelligence	5.5 ± 0.9	0.89
SEA	21.8 ± 4.2	0.75
OEA	23.4 ± 4.2	0.83
UOE	23.0 ± 4.3	0.83
ROE	20.3 ± 5.5	0.87
Job performance	4.0 ± 0.6	0.91

**Table 4:** Simple linear regression analyses of the factors associated with pharmacists’ job performance (*n* = 352).

Variable	B	Standard Error	β	t	95% Confidence Interval		*p*-Value
					Lower	Upper	
Emotional intelligence	0.43	0.03	0.58	13.18	0.36	0.49	<0.001 *
Age	0.12	0.15	0.04	0.77	−0.18	0.41	0.443
Gender							
Men	−0.18	1.12	−0.01	−0.16	−2.38	2.02	0.873
Women	Ref.						
Marital status							
Married	−0.15	1.12	−0.01	−0.13	−2.35	2.06	0.897
Non-married	Ref.						
Income in SAR/1000 (USD 0.266)	0.1	0.12	0.04	0.89	−0.13	0.34	0.374
Years of work experience	0.19	0.145	0.07	1.31	−0.10	0.47	0.191
Total working hours/week	−0.07	0.1	−0.04	−0.67	−0.26	0.13	0.504
Community pharmacist							
Yes	−1.53	1.1	−0.07	−1.40	−3.70	0.63	0.164
No	Ref.						

B: Unstandardized coefficients, β: Standardized coefficients, SAR: Saudi riyal, Ref: Reference, * statistically significant.

**Table 5:** Multiple linear regression analysis of the variables associated with pharmacists’ job performance (*n* = 352).

Variable	B	Standard Error	β	t	95% Confidence Interval		*p*-Value
					Lower	Upper	
Emotional intelligence	0.43	0.03	0.57	13.1	0.36	0.49	<0.001 *
Gender							
Men	0.05	0.93	0.002	0.06	−1.79	1.89	0.955
Women	Ref.						
Experience in years	0.15	0.12	0.05	1.21	−0.09	0.39	0.228
Community pharmacist							
Yes	−0.71	0.94	−0.03	−0.76	−2.56	1.14	0.449
No	Ref.						

B: Unstandardized coefficients, β: Standardized coefficients, Ref: Reference, * statistically significant.

**Table 6:** Correlation between job performance and emotional intelligence domains along with other independent variables.

Item	Correlation	*p*-Value
Emotional intelligence		
SEA	0.42	<0.001 *
OEA	0.31	<0.001 *
UOE	0.62	<0.001 *
ROE	0.40	<0.001 *
Age	0.04	0.4431
Gender	−0.01	0.8732
Marital status	−0.01	0.8971
Income in SAR (USD 0.266)	0.05	0.3736
Years of work experience	0.07	0.1911
Total working hours/week	−0.04	0.5041
Community pharmacist	−0.07	0.1637

SEA: self-emotion appraisal, OEA: others’ emotion appraisal, UOE: use of emotion, ROE: regulation of emotion, * statistically significant.

## Data Availability

The data disclosed in this study is obtainable upon request from the corresponding author.
